# Maintaining microendemic primate species along an environmental gradient – parasites as drivers for species differentiation

**DOI:** 10.1002/ece3.1311

**Published:** 2014-12-02

**Authors:** Simone Sommer, Solofomalla Jacques Rakotondranary, Jörg U Ganzhorn

**Affiliations:** 1 Evolutionary Genetics, Leibniz-Institute for Zoo- and Wildlife ResearchAlfred-Kowalke-Strasse 10, Berlin, 10315, Germany; 2 Institute for Experimental Ecology, University of UlmAlbert-Einstein Allee 11, Ulm, 89069, Germany; 3 Department of Animal Ecology and Conservation, University of Hamburg, Biozentrum GrindelMartin-Luther-King Platz 3, Hamburg, 20146, Germany

**Keywords:** Helminths, hybrids, lemurs, local adaptations, Madagascar, MHC, *Microcebus* spp., primates, species differentiation

## Abstract

Understanding the drivers of species adaptations to changing environments on the one hand and the limits for hybridization on the other hand is among the hottest questions in evolutionary biology. Parasites represent one of the major selective forces driving host evolution and at least those with free-living stages are at the same time dependent on the ecological conditions of their host's habitat. Local immunological adaptations of host species to varying parasite pressure are therefore expected and might represent the genetic basis for ecological speciation and the maintenance of recently diverged species. Madagascar provides one of the rare examples where two partially sympatric primate species (*Microcebus griseorufus, M. murinus*) and their hybrids, as well as an allopatric species (*M.* cf *rufus*) live in close proximity along a very steep environmental gradient ranging from southern dry spiny bush to gallery forest to evergreen eastern humid rain forest, thus mimicking the situation encountered during extensions and retreats of vegetation formations under changing climatic conditions. This system was used to study parasite infection and immune gene (MHC) adaptations to varying parasite pressure that might provide selective advantages to pure species over hybrids. Parasite burdens increased with increasing humidity. *M. griseorufus*, *M. murinus,* and their hybrids but not *M. rufus* shared the same MHC alleles, indicating either retention of ancestral polymorphism or recent gene flow. The hybrids had much higher prevalence of intestinal parasites than either of the parent species living under identical environmental conditions. The different representation of parasites can indicate a handicap for hybrids that maintains species identities.

## Introduction

Understanding the processes and mechanisms involved in shaping the ability of individuals to adapt to their local environment is a major avenue in evolutionary biology and conservation genetics. Parasites act as one of the main selective forces (Altizer et al. 2003). Under natural conditions individuals are permanently exposed to parasites. Especially, gastrointestinal helminths can reach high prevalence levels in their host populations and represent important evolutionary forces as they partly feed on mucosa cells, cause bleeding and reduce food intake (Lowrie et al. 2004). This in turn influences the fitness of individuals and can drive speciation (Buckling and Rainey 2002). Environmental conditions play an important role in the distribution, transmission, and developmental success of parasites (Mas-Coma et al. 2008). Thus, habitat-specific differences in biotic and abiotic conditions influence the presence and abundance of potential intermediate hosts as well as parasites and thus shape local parasite pressures (Kaltz and Shykoff 1998). This in turn should select for contrasting local immunogenetic adaptations of hosts that inhabit these different habitats (Eizaguirre and Lenz 2010; Lenz et al. 2013; Froeschke and Sommer 2014).

Genes of the major histocompatibility complex (MHC) play a key role in the host's adaptive immune response and are of central importance in parasite defense (Sommer 2005). They code for cell surface glycoproteins, which recognize and bind antigens derived from pathogens or parasites and present them to T lymphocytes, which in turn initiate the immune response (Klein et al. 1998). High levels of MHC polymorphism and evidence for positive selection is frequently observed in natural populations and considered as an adaptation to detect a wide array of rapidly evolving parasites and pathogens (Sommer 2005). Several evolutionary mechanisms have been suggested. The most debated ones refer to pathogen-driven selection by the effects of a “heterozygote advantage” (Doherty and Zinkernagel 1975), by the temporal advantage of specific alleles (“rare allele advantage hypothesis,” “negative frequency-dependent selection;” Clarke and Kirby 1966), by selection that varies in space or time due to local shifts in parasite pressure (“fluctuating selection,” Hill et al. 1994; Hedrick 2002), or by an “immunogenetic optimality” (Reusch et al. 2001; Wegner et al. 2003).

During the past decade, the ecological theory of adaptive speciation and radiation has received considerable attention. It assumes incipient speciation as a direct or indirect result of niche-based ecological differences and divergent selection pressure. Madagascar's biota are composed of many microendemic species. Particularly, the geographical ranges of lemurs are on average orders of magnitudes smaller than the ranges of other primate species, and the diversity of species exceeds the primate diversity in other areas of the world (Myers et al. 2000; Rasoloarison et al. 2000; Yoder et al. 2002; Mittermeier et al. 2010). Southeastern Madagascar provides one of the rare examples where closely related primate species, lemurs of the same genus, show striking pattern of sympatry and allopatry. As an example, two partially sympatric mouse lemur species (*Microcebus murinus, hereafter Mm* and *M. griseorufus, Mg*) and an allopatric species (*M. cf rufus, Mr*) live in close proximity along a very steep environmental gradient ranging from southern dry spiny bush to gallery forest (*Mg, Mm*) to evergreen humid rain forest (*Mr*). The three species show clear associations with the specific habitat types but distributions overlap at ecotones. Nevertheless, the ecotone between dry spiny and gallery forest represents a species boundary between *Mg* and *Mm* while the ecotone between dry spiny forest and evergreen humid forest represents the species boundary between *Mm* and *Mr*. So far, there are no reports of hybridization between *Mm* and *Mr*. *Mr* evolved within a lineage that split off from the *Mg* and *Mm* group ca. 5–12 million years ago, that is, much earlier than the divergence between *Mg* and *Mm* (Yoder and Yang 2004). Hybrids between *Mg* and *Mm* were observed; however, in disturbed areas, that is, at sites where dry and mesic habitats come into mosaic-type contact (Rakotondranary et al. 2011a; Hapke et al. 2011). Despite intensive research on the morphology, ecology, diet, behavior, and physiology of all species, with a specific focus on intraspecific competition and niche-based ecological differences (e.g., Ortmann et al. 1997; Schmid 2000; Yoder et al. 2002; Génin 2008; Gligor et al. 2009; Kobbe and Dausmann 2009; Schmid and Ganzhorn 2009; Rakotondranary et al. 2011a,b; Kobbe et al. 2011; Thoren et al. 2011), the mechanisms generating and maintaining these patterns of microendemisms and occurrence of hybrids remain enigmatic (Yoder et al. 2005; Wilme et al. 2006; Vences et al. 2009; Rakotondranary et al. 2011a; Zinner et al. 2011).

In order to understand the mechanisms that drive microevolutionary processes in sympatric congeneric species, we investigated three species of *Microcebus* (*Mg, Mm, Mr*) occurring along an environmental gradient. We used them as model to investigate local adaptations that might explain the sympatric and allopatric distribution of microendemic species and shed light on the selective advantages of pure species over hybrids. As habitat-specific variations in parasite pressure requiring local immunological adaptations of host species might represent the genetic basis for ecological speciation and the maintenance of recently diverged species, we investigated gastrointestinal helminth burden and the MHC constitution across habitats and species.

Our specific questions were as follows:

1. Are there differences in the parasite pressure along the environmental gradient ranging from dry spiny bush to evergreen humid forest?
2. Given the evolution of microendemics, do sympatric and allopatric microendemics differ in their immune genetic constitution?
3. Do microendemics have specific genetic adaptations to parasite pressure that lead to separation?

## Material and Methods

### Study area

**Figure 1:**
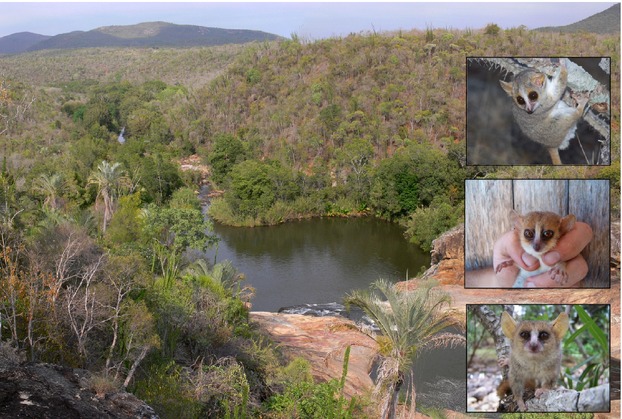
*Microcebus griseorufus* (white belly, top) occurs in the dry spiny forest, *Microcebus murinus* (gray belly, bottom) in the dry spiny and gallery forest with a small band of hybridization (Mg x Mm, middle) at the ecotone in Tsimelahy, Madagascar (photo W. Berg and S. J. Rakotondranary).

**Figure 2:**
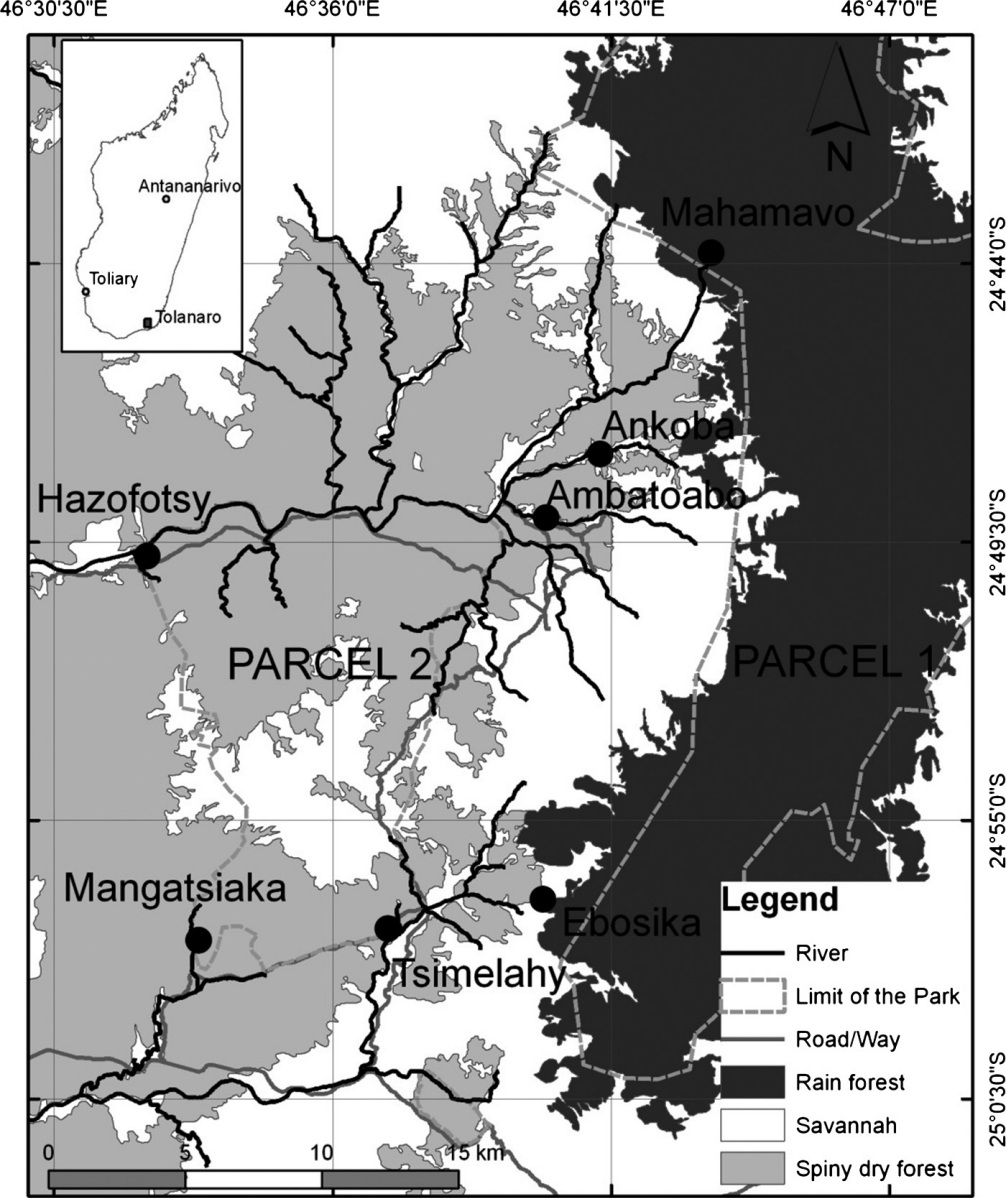
Study sites in the Andohahela National Park in southeastern Madagascar. Gallery forests are too narrow to be depicted in the figure. They stretch as a narrow band of just a few meters width along the rivers (modified from Google Earth and Rakotondranary et al. 2011a).

The study on the different forms of mouse lemurs (Fig. 1) was carried out at Andohahela National Park (Parcel 1, Parcel 2, and in the nonprotected area between Parcel 1 and 2) in southeastern Madagascar (Fig. 2). This area provides an ideal opportunity to investigate adaptive processes in the genus *Microcebus* because it consists of a continuous environmental gradient ranging from southern dry spiny forest with 400 mm/years rainfall to the eastern evergreen humid rain forest with a precipitation of 2400 mm/years (Barthlott et al. 1996; Goodman 1999). The environmental conditions (ambient temperature, humidity, plant species composition, phenology, and vegetation structure) vary systematically along the gradient and are described by Goodman (1999), Moat and Smith (2007), Andriaharimalala et al. (2011), and Rakotondranary et al. (2011a).

*Microcebus* individuals were caught at seven different sites along two transects from the dry west to the humid east covering the three types of vegetation (dry spiny forest DSF, gallery forest GF, and rain forest RF) (Fig. 2). The northern transect consisted of sites at Hazofotsy (DSF), Ambatoabo (DSF, GF), Ankoba (DSF), and Mahamavo (RF). The southern transect was composed of sites at Mangatsiaka (DSF, GF), Tsimelahy (DSF, GF), and Ebosika (DSF) (see Hapke et al. (2011) for detailed maps and habitat descriptions). The standardized trapping procedure, animal handling, and welfare protocol, as well as the morphological and ecological data collection are described in detail in Rakotondranary and Ganzhorn (2011). Briefly, trapping was performed with Sherman traps (7.7 × 7.7 × 23 cm) during the end of the dry season (September, October, and November) and at the end of the wet season (April, May, June) between September 2006 and June 2009. Traps were placed on a tree, spaced at 25 m intervals and baited with bananas for four successive nights per transect. From each individual, a small ear tissue sample (2 × 1 mm) and feces for gastrointestinal parasite screening were collected and stored in 80% ethanol. Species identity was verified, and hybrids identified by morphological features and genetic markers according to Rakotondranary et al. (2011a) and Hapke et al. (2011). Only adults were included in subsequent analyses.

### MHC diversity analyses

The DNA was isolated from ear biopsies following standard protocols (Qiagen QIAmp DNA Mini Kit No. 51306, Hilden, Germany). We focused our analyses on the highly polymorphic second exon of MHC class II DRB because this region includes the functionally important antigen-binding sites (ABS), that is, amino acid positions postulated to interact directly with foreign antigens derived from extracellular parasites and pathogen (e.g., gastrointestinal helminths) (Brown et al. 1993). We used the primers Migr.DRB Intron1 3′YCCTCCGYGTCCCCACAG5′ and Tub2JS 3′GATCCCGTAGTTGTGTCTGCA5′ which successfully amplified a 219-bp product in all 171 investigated individuals. The 25 *μ*L reactions contained 1 *μ*L of extracted genomic DNA (100 ng/*μ*L) in a final concentration of 1× buffer (Roche), 2 mmol/L MgCl_2_, 0.5× GC-Rich Solution, 0.2 mmol/L dNTPs (Roche), 0.4 *μ*mol/L of each primer, and 0.5 U Taq-Polymerase (Fast Start Taq, Roche). Cycling conditions consisted of 15 min initial denaturation at 95°C followed by 38 rounds of 30 sec denaturation at 95°C, 60 sec annealing at 62°C, and 60 sec extension at 72°C, and a final 7 min extension at 72°C after the last cycle. PCR was performed on a T-Gradient Thermocycler 96 (Biometra, Göttingen). Negative controls were always included to check for contamination. For verification of successful amplification, 5 *μ*L of PCR product was visualized in ethidium-bromide-stained 2.0% agarose gels. The MHC class II-DRB genotyping was conducted through single-stranded conformation polymorphism (SSCP) analysis followed by Sanger sequence analysis of the re-amplified distinctive single-strand bands as previously described (Schad et al. 2005; Schwensow et al. 2010a,b).

### Parasites screening

Fecal egg counts were conducted using a modification of the widely used McMaster technique, an easy, noninvasive, and suitable technique to gauge the intensity of helminth infections (Soulsby 1982; Sloss et al. 1994). Fecal samples were screened for helminth eggs by counting two chambers of the McMaster slide using a light optical microscope. Instead of the conventional saturated sodium chloride solution with a specific weight of 1.2 g/mL, we used potassium iodide with a specific density of 1.5 g/mL (Meyer-Lucht and Sommer 2005) to enhance the detectability of eggs with high specific weights, such as some nematode and trematode eggs (Thienpont et al. 1986). This method has been used in a number of recent studies (e.g. Meyer-Lucht and Sommer 2005; Schad et al. 2005; Axtner and Sommer 2007; Schwensow et al. 2007; Froeschke et al. 2010). It has been shown that the number of nematode eggs and larvae found in the feces correlates very well with the intensity of infection, that is, worm burden in the digestive tract of mouse lemurs (Raharivololona 2006). We classified helminth eggs according to Raharivololona (2006), Raharivololona et al. (2007), and Raharivololona (2009) based on size and appearance and photographed them for later taxonomic identification. We assessed the prevalence and the intensity of the different helminth infections by calculating the number of nematode eggs per gram feces (EPG).

### Data analyses and statistical treatment

We used MEGA 5 (Tamura et al. 2011) to align and edit the MHC DRB sequences and to calculate the number of constant and variable sites, the mean number of nucleotide and amino acid differences, as well as the genetic distances between species. We investigated signals of positive selection on the MHC antigen-binding sites by calculating the relative rates of nonsynonymous (*d*_N_) and synonymous (*d*_S_) base pair substitutions according to Nei and Gojobori (1986) applying the Jukes–Cantor correction for multiple hits (Jukes and Cantor 1969). We carried out all calculations separately for putative antigen-binding sites (ABS) and non-ABS assuming concordance of the lemur side chain residues with the human ABS (Brown et al. 1993, 1988). To test for differences of the *d*_N_/*d*_S_ rates, we used a two-sided Z-test implemented in MEGA 5. FSTAT ver 2.9.3 (Goudet 2001) was applied to calculate allelic richness and *F*_IS_-values. Departures from Hardy–Weinberg equilibrium were assessed using Arlequin ver 3.5. (Excoffier and Lischer 2010).

The software Quantitative Parasitology 3.0 (Rózsa et al. 2000) was used to calculate the prevalence (number of infected individuals) and the median intensity (median number of parasites found in infected hosts with the zeros of uninfected hosts excluded), both with confidence intervals. Parasite individuals typically exhibit an aggregated (right-skewed) distribution among host individuals with most hosts harboring few if any parasites and a few hosts harbor most of them. The median intensity shows a typical level of infection among the infected hosts and is not affected by the few highly infected host individuals (Rózsa et al. 2000). Differences between prevalence and infection intensity were tested by Fisher's exact tests and Mood's median test, respectively, both implemented in Quantitative Parasitology 3.0.

We analyzed whether specific MHC alleles have a significant influence upon parasite burden using generalized linear modelling (GLMs) as recommended by O'Hara and Kotze (2010) and described by Schwensow et al. (2010a,b) and Axtner and Sommer (2012). We restricted our analyses to parasites with a prevalence in the overall data set larger than 10%. We corrected for overdispersion using the quasipoisson family (Crawley 2007; O'Hara and Kotze 2010). We started with the full model including all predictors (i.e. MHC alleles present in at least five individuals) and conducted backward selection. We selected the most parsimonious models by dropping insignificant terms sequentially from the model until only significant terms were left or the model deviance grew significantly higher. We compared model deviance between sequential models using the ANOVA function of R. We calculated the adjusted *R*^2^ value as 1- (model deviance/model df)/(null model deviance/null model df). All statistical analyses were conducted using R version 2.13 (R development Core Team 2011) or SPSS vers 18.0. Whenever the data fulfilled the requirements, we used parametric tests, otherwise we applied nonparametric tests. Calculations are based on a significance level of *α* = 0.05.

## Results

### Comparison of MHC diversity in *Microcebus* spp.

The individual MHC class II DRB exon 2 diversity was successfully genotyped in all captured 171 *Microcebus* individuals (39 *Mg*, 97 *Mm*, 17 hybrids (*Mg* × *Mm*) and 18 *Mr,* Fig. 2, Table 1). In total, 92 different MHC alleles were detected. No more than two alleles were observed per individual, suggesting that a single locus was amplified. Twenty-seven new *Mg* sequences (*Migr*-DRB*1 to *27, KF183517–KF183543), 41 new *Mm* sequences (*Mimu*-DRB*71 to *111, KF183544–KF183584), and 24 new *Mr* sequences (*Miru*-DRB*1 to *24, KF183585–KF183608) were submitted to GenBank.

**Table 1:** Habitat and site specificity of *Microcebus griseorufus* (*Mg*), *M. murinus* (*Mm*), hybrids (*Mg* × *Mm*), and *M. cf rufus* (*Mr*). The number of individuals trapped in the dry spiny forest (DSF), gallery forest (GF), and rainforest (RF) are indicated. Unused habitats/sites are marked in gray. *N* overall sample size

	*N*	Hazofotsy	Mangatsiaka		Tsimelahy		Ebosika	Ankoba	Ambatoabo		Mahamavo
Habitat		DSF	DSF	GF	DSF	GF	DSF	DSF	DSF	GF	RF
*Mg*	39	15	8		15		1				
*Mm*	97		33 (3)	18 (3)	9	10	1	5	10	8	
*Mg* × *Mm*	17	1	10 (1)	2 (1)	1		1			1	
*Mr*	18										18

Four *Mimu* alleles were previously detected in *Mm* individuals trapped in the dry deciduous Kirindy forest at the western coast of Madagascar, ca. 500 km far away from this study site (Mimu*19 EU137063, Mimu*42 EU137086, Mimu*44 EU137088, Mimu*62 HE801956; Schwensow et al. 2010a,b; Huchard et al.*,* 2012). Two *Mimu* alleles were previously reported from the nearby Mandena littoral forest in southeastern Madagascar, ca. 40 km apart (Mimu*9 AJ555838, Mimu*6 AJ431270; Schad et al. 2005,2012).

**Figure 3:**
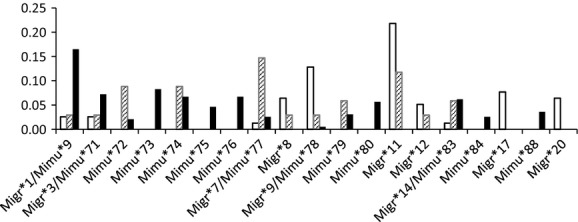
Relative frequencies of MHC class II DRB alleles present in at least five individuals. *Mg* white bars; hybrids hatched bars, *Mm* black bars.

In Andaohela, the two species *Mg* and *Mm* shared six MHC alleles (Migr*1/Mimu*9, Migr*3/Mimu*71, Migr*7/Mimu*77, Migr*9/Mimu*78, Migr*14/Mimu*83, Migr*15/Mimu*85), whereas *Mr* carried only unique species-specific MHC alleles. The MHC alleles detected in hybrid individuals were either unique to the hybrids (five alleles), or also detected in *Mg*, *Mm* or in both species (5, 6, and 5 alleles, respectively). The frequencies of shared alleles differed up to 13% between *Microcebus* spp. (Fig. 3). All pairwise *F*_ST_ values between *Microcebus* spp. were significant (*F*_ST_: 0.02–0.06, all *P* < 0.01).

All species exhibited high levels of heterozygosity which differed from Hardy–Weinberg expectations in *Mm* and the hybrids although the departures were relatively minor. The amino acid distance between individual MHC alleles was higher in *Mr* than in the other species (*Mg*: 12.5 ± 5.7, hybrid: 12.2 ± 6.1, *Mm*: 11.1 ± 5.6, *Mr*: 14.9 ± 5.9; Anova: *P* = 0.055, df = 3, Bonferroni post hoc test: *P* < 0.05). All species showed high levels of allelic richness, number of conserved and variable sites, as well as genetic distance measurements (Table 2). Thereby, the allelic richness was significantly higher in *Mr* and significantly lower in *Mg* than in all the other species (all *P* < 0.001). All species showed clear signs of positive selection (Table 3). Noteworthy, the nonsynonymous substitution rate in antigen-binding sites was significantly higher in hybrids than in all three pure species (Anova: *n* = 5.183, df = 3, *P* < 0.001, Tukey's post hoc tests: hybrid-Mg: *P* = 0.002, hybrid-Mm: *P* = 0.021, hybrid-Mr: *P* = 0.004).

**Table 2:** MHC diversity indices observed in *Microcebus griseorufus* (*Mg*), *M. murinus* (*Mm*), their hybrids (*Mg* × Mm) and *M. cf rufus* (*Mr*). The number of genotyped individuals (*N*), number of observed MHC alleles, observed and expected heterozygosities according to Hardy–Weinberg expectations (HWE), number of conserved (C) and variable (V) sites, as well as the genetic distance and number of differences on the nucleotide and amino acid level are indicated

Species	*N*	# Alleles	Allelic Richness	*H*_obs_/*H*_exp_	*P* (HWE)	*F*_IS_	C	V	Mean NuclDist	Mean AADist	Mean Nr NuclDiff	Mean Nr AADiff
Mg	39	27	17.2	0.90/0.92	0.09	0.027	165	51	0.10 + 0.01	0.21 + 0.04	20.32 + 2.66	13.92 + 2.44
Hybr	17	20	20.0	0.88/0.96	0.01	0.079	165	54	0.11 + 0.02	0.23 + 0.05	21.72 + 2.85	14.85 + 2.32
Mm	97	43	18.2	0.87/0.94	0.03	0.080	157	62	0.10 + 0.01	0.22 + 0.04	20.80 + 2.59	14.32 + 2.22
Mr	18	23	22.2	0.94/0.97	0.50	0.029	163	56	0.11 + 0.02	0.23 + 0.04	21.47 + 2.85	15.00 + 2.35

**Table 3:** The estimated rates (±standard error) of nonsynonymous (*d*_N_) and synonymous (*d*_S_) substitutions for antigen (ABS) and nonantigen (non-ABS) binding sites (ABS according to the human sequence, Brown et al. 1988, 1993), and their ratio for MHC class II exon 2 sequences in *Mg, hybrids Mg* × *Mm, Mm, and Mr*. N is the number of codons in each category and *P* is the probability that *d*_N_ equals *d*_S_ using a two-sided Z-test

Mg	*N*	*d*_N_	*d*_S_	*d*_N_/*d*_S_	*P*
ABS	18	0.44 ± 0.10	0.03 ± 0.02	12.9	<0.0001
Non-ABS	74	0.05 ± 0.02	0.03 ± 0.02	1.7	ns
All	92	0.12 ± 0.02	0.03 ± 0.01	4.4	<0.0001

Hybrids	*N*	*d*_N_	*d*_S_	*d*_N_/*d*_S_	*P*
ABS	18	0.50 ± 0.11	0.05 ± 0.03	10.7	<0.0001
Non-ABS	74	0.05 ± 0.02	0.02 ± 0.02	2.2	ns
All	92	0.14 ± 0.03	0.03 ± 0.01	4.8	<0.0001

Mm	*N*	*d*_N_	*d*_S_	*d*_N_/*d*_S_	*P*
ABS	18	0.44 ± 0.08	0.05 ± 0.03	9.1	<0.0001
Non-ABS	73	0.05 ± 0.02	0.03 ± 0.02	1.6	ns
All	92	0.13 ± 0.02	0.03 ± 0.02	3.7	<0.0001

Mr	*N*	*d*_N_	*d*_S_	*d*_N_/*d*_S_	*P*
ABS	18	0.42 ± 0.08	0.03 ± 0.02	12.5	<0.0001
Non-ABS	73	0.05 ± 0.02	0.04 ± 0.02	1.3	ns
All	92	0.13 ± 0.02	0.04 ± 0.02	3.3	0.002

### Habitat specificity of parasite loads

*Mg* only occurred in the dry spiny forests of Hazofotsy, Mangatsiaka, Tsimelahy, and Ebosika. *Mm* were caught in the dry spiny and gallery forest sites of Ankoba, Ambatoabo, Mangatsiaka, Tsimelahy, and Ebosika. The hybrids were trapped in the dry spiny and gallery forest sites of Hazofotsy, Ambatoabo, Mangatsiaka, Tsimelahy, and Ebosika. *Mr* were only present in the rainforest site Mahamavo. Four individuals moving between the dry spiny and the gallery forest were excluded from habitat-specific analyses (Fig. 2, Table 1).

In 170 *Microcebus* fecal samples (38 *Mg*, 97 *Mm*, 17 hybrids, and 18 *Mr*), we distinguished twelve different types of helminth eggs (cestodes, nematodes) (Table 4). As identification of helminth eggs to the species level is uncertain, we used the conservative approach and assigned the eggs to morphotypes. We distinguished two cestode egg morphotypes belonging to the family of *Hymenolepididae* (*Hymenolepis* sp.). Of the ten nematode morphotypes, one belonged to the family of *Ascaridae* (*Ascaris* sp.), five could not further classified (nematodes 1–5), and four were members of the family *Oxyuridae* (*Lemuricola* sp., Oxyurids 1–3). The overall helminth prevalence ranged from 0.01 through 0.13 with *Hymenolepis1* and *Ascaris* being the most prevalent infections (>10% of all individuals, Table 4).

**Table 4:** Helminth prevalence and 95% confidence intervals (CI) detected in 170 Microcebus fecal samples (38 *Mg*, 97 *Mm*, 17 hybrids *Mg* × *Mm* and 18 *Mr*). ns *P* > 0.05, na not applicable. Parasites are described in detail, and pictures provided in Raharivololona et al. (2007) and Raharivololona (2009)

	Overall		*M. griseorufus*		*Hybrids*		*M. murinus*		*M. cf rufus*		Fisher's
						
	Prev	CI	Prev	CI	Prev	CI	Prev	CI	Prev	CI	Exact Test
Cestoden											
Hymenolepis1	0.13	0.08–0.19	0.03	0.00–0.14	0.12	0.02–0.35	0.08	0.04–0.15	0.61	0.38–0.82	**<0.0001**
Hymenolepis2	0.01	0.00–0.03	–	–	–	–	–	–	0.06	0.00–0.27	na
Nematodes											
Ascaris	0.11	0.07–0.16	–	–	0.18	0.05–0.42	0.08	0.04–0.15	0.39	0.19–0.63	**<0.0001**
Nematode1	0.08	0.05–0.14	0.11	0.04–0.25	0.12	0.02–0.35	0.08	0.04–0.15	–	–	ns
Nematode2	0.01	0.00–0.04	–	–	0.06	0.00–0.29	–	–	0.06	0.00–0.27	**0.041**
Nematode3	0.01	0.00–0.03	–	–	–	–	0.01	0.00–0.06	–	–	na
Nematode4	0.03	0.01–0.07	–	–	0.06	0.00–0.29	0.04	0.01–0.10	–	–	ns
Nematode5	0.01	0.00–0.04	–	–	–	–	0.02	0.00–0.07	–	–	na
Lemuricola	0.02	0.01–0.06	–	–	–	–	0.04	0.01–0.10	–	–	na
Oxyurid1	0.01	0.00–0.03	–	–	–	–	0.01	0.00–0.06	–	–	na
Oxyurid2	0.01	0.00–0.04	–	–	–	–	0.02	0.00–0.07	–	–	na
Oxyurid3	0.01	0.00–0.04	–	–	0.06	0.00–0.29	0.01	0.00–0.06	–	–	ns

Significant Fisher's Exact tests (*P* < 0.05) are Marked in bold.

In the overall data set (*n* = 170), the nematode prevalence and infection intensity did neither differ between sampling years nor seasons (September, October, November vs. April, May, June) (Kruskal–Wallis tests: all *P* > 0.14). Also, the burden of the nematodes *Ascaris* and Nematode 1 was neither affected by year nor season (Kruskal–Wallis tests: all *P* > 0.36). The cestode prevalence and infection intensity tended to differ between sampling years (Kruskal–Wallis tests: *P* = 0.059 and 0.053, respectively) and were higher at the end of the rainy season (Kruskal–Wallis tests: *P* = 0.020 and 0.018, respectively). Accordingly, the burden of the most frequent cestode *Hymenolepis1* was affected by year and season (Kruskal–Wallis tests: all *P*: 0.008–0.025).

**Figure 4:**
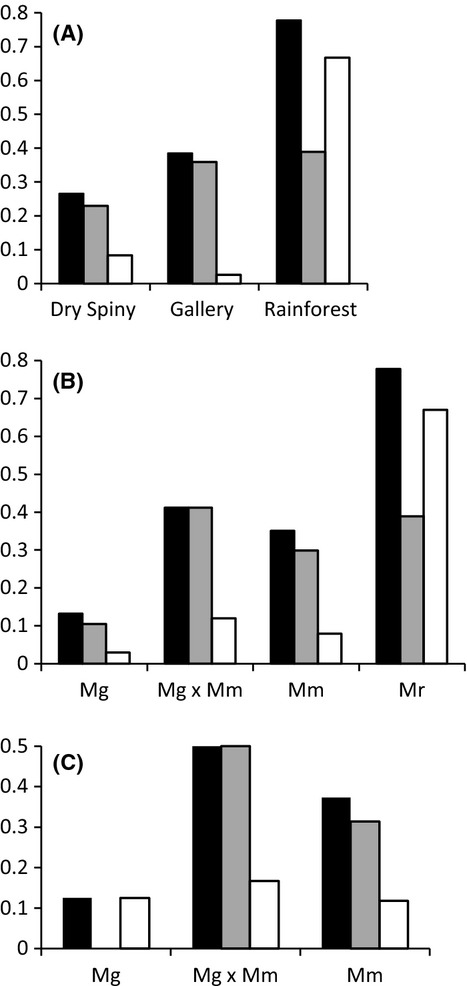
Prevalence of helminths (black bars), nematodes (gray bars), and cestodes (white bars) in (A) different habitats, (B) *Microcebus* spp. (*Microcebus griseorufus Mg*, hybrids *Mg* × *Mm*, *M. murinus Mm*, *M. cf rufus Mr*), and (C) sympatric *Mg*, hybrids, and *Mm* in the major hybrid zone Mangatsiaka.

Individuals caught in different habitats differed in their parasite burden which was highest in rainforest individuals and lowest in dry spiny forest individuals except for cestodes which showed the lowest value in the gallery forest (all helminths: prevalence: Fisher's exact test *P* < 0.0001, infection intensity: Mood's median test *P* = 1.00; nematodes: Fisher's exact test *P* = 0.146, Mood's median test *P* = 0.019; *Ascaris*: Fisher's exact test *P* = 0.002; Mood's median test *P* = 1.00, cestodes: Fisher's exact test *P* < 0.0001, Mood's median test *P* = 1.00; *Hymenolepis1*: Fisher's exact test *P* < 0.0001, Mood's median test *P* = 1.00; Fig. 4A).

### Host-specific patterns of helminth parasites

The *Microcebus* species showed marked differences in helminth burden (all helminths: Fisher's exact test *P* < 0.0001, Mood's median test *P* = 0.004; nematodes: Fisher's exact test *P* = 0.025, Mood's median test *P* = 0.033; *Ascaris*: Fisher's exact test *P* < 0.0001; Mood's median test *P* = 1.00, cestodes: Fisher's exact test *P* < 0.0001, Mood's median test *P* = 0.822; *Hymenolepis1*: Fisher's exact test *P* < 0.0001, Mood's median test *P* = 0.817; Fig. 4B). In relation to their habitat preferences, helminth prevalence was highest in the rainforest species *Mr*. But varying parasite pressure due to climatic conditions in different habitats cannot explain differences in the other *Microcebus* spp. since *Mg*, hybrids and *Mm* co-occur in the dry spiny forest.

The overall helminth burden was higher in hybrids than in both parent species *Mg* and *Mm* (Fisher's exact test *P* = 0.020, Mood's median test *P* = 0.701). Hybrids were most infected by nematodes (Fisher's exact test *P* = 0.019, Mood's median test *P* = 0.876). This was mainly driven by the nematode Ascaris (Fisher's exact test *P* = 0.034, Mood's median test *P* = 1.000, Table 4), whereas the overall cestode as well *Hymenolepis1* burden did not differ between species (cestodes and *Hymenolepis1*: Fisher's exact test *P* = 0.349, Mood's median test *P* = 0.697) (Fig. 4B).

In order to exclude any possible confounding effect of habitats, we restricted the interspecific comparison to the captures at Mangatsiaka, a site where both parent species and their hybrids occur sympatrically (Fig. 2, 4C). Hybrids had the highest helminth, nematode, and cestode prevalence, and again differences were mainly driven by nematodes (all helminths: prevalence: Fisher's exact test *P* = 0.282, infection intensity: Mood's median test *P* = 1.00; nematodes: Fisher's exact test *P* = 0.043, Mood's median test *P* = 1.00; cestodes: Fisher's exact test *P* = 0.851, Mood's median test *P* = 1.00, Fig. 4C). In Mangatsiaka, *Mg* were not infected by nematodes at all.

Comparison of the nematode burden of individuals trapped in the two different habitats (dry spiny forest, gallery forest) in Mangatsiaka showed that in the dry spiny forest the nematode prevalence was highest in the hybrid individuals (*Mg*: x: 0.000, CI 95%: 0.000–0.365; *Mg × Mm*: x: 0.500, CI 95%: 0.223–0.778; *Mm*: x: 0.212, CI 95%: 0.102–0.378; Fisher's exact test: *P* = 0.042, Mood's median test *P* = 1.00). Hybrid individuals showed no differences in prevalence between dry spiny and gallery forest. In the gallery forest, hybrids and *Mm* had similar levels of nematode infections (*Mg* × *Mm*: x: 0.500, CI 95%: 0.025–0.975; *Mm*: x: 0.500, CI 95%: 0.271–0.729). The nematode prevalence in *Mm* tended to be higher in the gallery than in the dry spiny forest (Exact *P*-value: 0.057, median test *P* = 1.00).

### Effect of MHC constitution in parasite burden

We investigated the general effect of specific MHC alleles on parasite burden. We included only adult individuals from which both genetic and fecal samples were available (*N* = 169). To avoid overparameterization, investigations on the relationship between MHC diversity and parasite load were restricted to 19 MHC alleles present in at least five individuals (Fig. 3). None of them occurred in *Mr*. All other alleles were merged to a single group of “rare alleles”. We included parasites with a prevalence in the overall data set larger than 10% (*Hymenolepis1*, *Ascaris*, Table 5).

**Table 5:** Effects of the specific MHC alleles on the infection intensity caused by the main gastrointestinal helminths *Ascaris* spp. and *Hymenolepis*1 of *Microcebus* spp. revealed by generalized linear models

*Ascaris* spp. ∼	Estimate	SE	*t* value	*P*
Intercept	**−**5.986	1.857	**−**3.224	0.002
Migr*1/Mimu*9	2.593	0.947	2.739	0.007
Mimu*75	3.100	1.098	2.824	0.005
Migr*7/Mimu*77	2.658	1.180	2.252	0.026
Mimu*80	5.302	0.856	6.197	<0.001
Migr*14/Mimu*83	3.483	1.219	2.856	0.005
Rare alleles	6.130	1.267	4.840	<0.001
Adjusted *R*^2^		0.585		
Null deviance:		6506.7	150 df	
Residual deviance:		2591.1	144 df	

*Hymenolepis1* ∼	Estimate	SE	*t* value	*P*
Intercept	−3.254	1.699	−1.915	0.057
Mimu*76	4.284	1.064	4.026	<0.001
Mimu*80	3.014	1.420	2.123	0.036
Rare alleles	3.571	1.487	2.401	0.018
Adjusted *R*^2^		0.553		
Null deviance:		2773.1	150 df	
Residual deviance:		1214.2	147 df	

*Ascaris* infection was associated with the presence of the alleles Migr*1/Mimu*9, Mimu*75, Migr*7/Mimu*77, Mimu*80, and Migr*14/Mimu*83 (Table 5). Migr*1/Mimu*9, the most frequent allele in the overall data set (10.2%), was 6× more frequent in *Mm* (16.5%) than in *Mg* and the hybrids. The frequency of Migr*7/Mimu*77 (overall: 3.2%) was 6.7× higher in hybrids (14.7%) than in *Mg* and Mm. Migr*14/Mimu*83 (overall: 4.4%) was 4× more frequent in hybrids and *Mm* (ca. 6.0%) than in *Mg*. Mimu*75, and *80 were only detected in *Mm* (Fig. 3). The same alleles also explained in a highly similar pattern overall nematode infections (Intercept: *P* = 0.004, Migr*1/Mimu*9: *P* = 0.016, Mimu*75: *P* = 0.014, Migr*7/Mimu*77: *P* = 0.046, Mimu*80: *P* < 0.001, Migr*14/Mimu*83: *P* = 0.013, rare alleles: *P* < 0.001).

Mimu*76 and Mimu*80, both detected in *Mm* only, were associated with *Hymenolepis1* infections (Table 5) and also explained overall cestode infections with highly similar values (Intercept: *P* = 0.057, Mimu*76: *P* < 0.001, Mimu*80: *P* = 0.035, rare alleles: *P* = 0.017). Including year/season in the models had no effect on the outcome. Limiting the analyses to MHC alleles present in more than 5% of the individuals did not change the results. The results were also not affected by including the amino acid distance between individual MHC alleles as a functional measurement of dissimilarity of heterozygous individuals or not.

In species-specific models for *Mg* or hybrids, no model fitted the data better than the null model probably due to sample size limitations. Models restricted to *Mm* supported the importance of allele Mimu*80 in *Ascaris* infections (Intercept: *P* = 0.071, Mimu*80: *P* = 0.004) and Mimu*76 and Mimu*80 in *Hymenolepis1* infections (Intercept: *P* = 0.807, Mimu*76: *P* < 0.001, Mimu*80: *P* = 0.027).

## Discussion

Parasites are considered as a major selective force driving evolution while they themselves are dependent on the ecological conditions of a given habitat. The idea that parasites play a significant role in sympatric and allopatric host diversification and can act as major drivers in ecological speciation has been discussed for decades (Mayr 1963; Hamilton and Zuk 1982; Haldane 1992). According to the theory, reproductive isolation (i.e., barriers to gene flow) evolves between sympatric populations due to divergent selection and adaptations to contrasting environments even in the absence of geographical barriers (e.g., Bolnick and Fitzpatrick 2007; Ritchie 2007). However, evidence for the role of parasites in maintaining species identity in natural populations is limited, and its mechanisms and genetic basis are still under investigation (Schluter 2001; Buckling and Rainey 2002; Eizaguirre et al. 2009; Abbott et al. 2013). Parasites can affect the fitness of their hosts through their energy budget (Devevey et al. 2008). Parasites increase host's energetic expenditure both directly by higher nutrient and energy demands and indirectly by increasing behavioral activity and triggering immune responses (Moore 2002). The energetic costs of parasitism lead to the altered allocation of available energy and ultimately to reduced growth, fecundity, and survival (Burns et al. 2005). Hybrids might be more susceptible to infection than their parental species due to genomic incompatibilities in the introgressed genomes of the hybrids (Sage et al. 1986; Moulia et al. 1991; Šimková et al. 2013). Also, extrinsic factors might play a role. Hybrids which live in intermediate environment or migrate among environments might be exposed to parasite communities infecting both parental species.

We found clear differences in the parasite pressure along the environmental gradient. The rainforest species *Mr* had the highest helminth prevalence and infection intensity, whereas individuals living in the dry spiny forest where mouse lemurs reach their ecological limits and are limited by food availability (Bohr et al. 2011) carried the lowest parasite burden or were not infected at all. As illustrated by other studies, microclimatic conditions and especially precipitation have a significant effect on the population dynamics of helminths (e.g., Nwosu 2007; Froeschke et al. 2010; Schwitzer et al. 2010). High ambient temperatures and humid conditions favor hatching of parasite eggs which increases the abundance of larvae ready to infect the next host (Larsen and Roepstorff 1999). The parasite development in intermediate insect hosts is also faster at higher temperatures (Pascual et al. 2006). Thereby, the parasites detected in mouse lemurs inhabiting Andohahela National Park (present study) were very similar to the detected helminth community in the Mandena forest, some 40 km east of the study area in Andohahela. All helminths have been described, including their seasonal occurrence in fecal samples, and illustrated in detail (Raharivololona 2006, 2009; Raharivololona et al. 2007; Raharivololona and Ganzhorn 2010). The majority of intestinal parasites infecting mouse lemurs are nematodes belonging to *Ascarididae*, *Strongylida*, *Trichuridae,* and *Oxyuridae*, as well as cyclophyllidean cestode species (reviewed by Irwin and Raharison 2009). The most abundant helminthes in our study were the nematode *Ascaris* sp. which has a direct life cycle and a cyclophyllidean cestode *Hymenolepis* spp. with an indirect life cycle. Both are transmitted through feces. The mouse lemurs probably get infected when sharing sleeping sites or when they descent to the ground to catch invertebrates. Mouse lemurs share many parasites with rats which move between disturbed areas and forest sites and rats represent a significant vector to native small mammal species, especially for *Ascaris* infections (Raharivololona 2006). The other prevalent nematode species have an indirect life cycle mainly using insects as intermediate hosts. Eggs develop into larvae which then are ingested by insect-feeding mouse lemurs (Irwin and Raharison 2009).

However, environmental variables cannot explain differences in the sympatric *Microcebus* spp. in the main hybrid zone where hybrids were significantly higher infected than both species of origin (*M. griseorufus*, *M. murinus*). Especially, the nematode *Ascaris* played a predominant role in hybrids. The importance of *Ascaris* infections is largely due to their impact on the nutritional status of the host, including protein, energy, and micronutrient malnutrition. This has been found to have significant fitness effects, for example, on the growth and the cognitive development, on the hemoglobin level and on pregnancy outcomes (reviewed by Crompton and Nesheim 2002).

Parasite-driven disruptive selection requires genetic variation in host populations which is affected by parasite pressure. Under these conditions, immunological adaptations of host populations to local parasite pressure are expected (Altizer et al. 2003; Sommer 2005; Lenz et al. 2013). On the other hand, these local genetic adaptations should be involved in reproductive isolation. The highly polymorphic MHC genes, key component in the adaptive immune system (Janeway et al. 2001), are ideal candidates to orchestrate parasite-driven species differentiation (Eizaguirre et al. 2009; Matthews et al. 2010). The pools of MHC alleles in different host populations and species are shaped by habitat-specific parasites (e.g., Blais et al. 2007; Schwensow et al. 2010a,b; Eizaguirre et al. 2012; Froeschke and Sommer 2014), and tight associations between specific MHC alleles and parasite burden have been demonstrated in many taxa (Sommer 2005). MHC genes also play a crucial role in mate choice (Penn and Potts 1999) and the individual MHC constitution affect fitness relevant trade-offs between host life history and immune defense as suggested by the “good genes” model (Schad et al. 2012). However, despite many fitting pieces of the puzzle, the role of parasites and immune gene diversity in host speciation is highly understudied (Summers et al. 2003b; Matthews et al. 2010), and so the consequences of parasite-mediated selection for host diversification remain uncertain (Nuismer et al. 2008; Eizaguirre et al. 2009).

In all *Microcebus* spp. as well as the hybrids, we found strong evidence for positive selection driving MHC diversity. Sympatric and allopatric microendemics as well as the hybrids showed high levels of allelic richness (17.2–22.2), heterozygosity (0.87–0.94), and individual allelic divergence in terms of genetic distance and number of substitutions between individual alleles. These are strong indications that the investigated loci in all species are involved in long-term host–parasite coevolutionary processes. Empirical studies have supported theoretical predictions that at a state of evolutionary equilibrium between hosts and parasites, that is, if hosts and pathogens share a long-term coevolutionary history (1st scenario), selection through diverse parasites cause high MHC polymorphism in a species or population, whereas low MHC polymorphism indicates the presence of relaxed pathogenic selection pressure (Goüy de Bellocq et al. 2008; Prugnolle et al. 2005; Wegner et al. 2003). However, in a recently introduced unbalanced situation due to anthropogenic disturbance, that is, after a recent loss of genetic diversity through limitation to gene flow (e.g., by habitat destruction or reproductive isolation, 2nd scenario), species with low MHC diversity could have lost resistance alleles or other important parts of its adaptive evolutionary potential which would facilitate an easy spread of parasites throughout the population (Meyer-Lucht and Sommer 2009). In accordance with the 1st scenario, the allopatric rainforest species *Mr* carrying the highest parasite burden revealed the highest allelic richness and heterozygosity, whereas *Mg* revealed the lowest allelic richness. In addition, the amino acid distance between individual alleles was significant larger in *Mr* than in the other species. Moreover, *Mr* carried a very distinct MHC allele repertoire. None of this species' alleles were detected in the two other mouse lemur species *Mm* and *Mg* living less than 20 km apart. This is noteworthy as some mouse lemur alleles had also been found in populations in Madagascar about 500 km apart. Contrarily to *Mr*, the two sympatric species as well as the hybrids shared MHC alleles. This phenomenon is described as trans-species polymorphism which indicates that alleles are older than the speciation event and were passed on from the ancestral to the descendant species due to balancing selection driven by parasites (Klein et al. 1998). One might argue that *Mr* evolved within a lineage that split off from the *Mg* and *Mm* group ca. 5–12 million years ago, that is, much earlier than the divergence between *Mg* and *Mm* (Yoder and Yang 2004). However, this earlier species differentiation does not explain the missing evidence for trans-species evolution in *Mr* as it has been shown to occur in many older taxa, such as in the genera *Rattus* and *Mus* which diverged at least 15 million years ago *(*Kumar and Hedges 1998; Musolf et al. 2004). This suggests that the selection pressure acting on allopatric *Mr* was always very distinct from the other sympatric mouse lemur species. Divergent parasite pressure might be one explanation why hybrids occur between *Mg* and *Mm* but were not detected (until now) between *Mr* and other *Microcebus* spp. Another, nonadaptive explanation for the lack of hybrids between *Mm* and *Mr* could be that genomic incompatibilities accumulated in allopatry after the divergence of *Mr*. For the time being, we cannot distinguish between these alternatives.

Do hybrids differ in their immunogenetic constitution? The MHC alleles detected in hybrid individuals were either unique to the hybrids (five alleles), or also detected in *Mg*, *Mm* or in both species (5, 6, and 5 alleles, respectively). In sympatric *M. murinus* as well as in the hybrids, we detected specific MHC alleles associated with *Ascaris* sp. and *Hymenolepis* sp. which might explain individual differences in parasite burden. Evidence that *Ascaris* exerts strong pressure on coevolutionary processes in the host MHC has already been indicated in a recent study in *Mm* and fat-tailed dwarf lemurs (*Cheirogaleus medius*) (Schwensow et al. 2010a,b). In their study, one specific MHC allele was identified in each species which was positively associated with *Ascaris* infection. Interestingly, these MHC alleles were very similar to each other but differed from all other investigated MHC alleles in an amino acid substitution in a putative functional important antigen-binding site. Thus, the study provided evidence for a direct connection between certain antigen-binding sites of MHC molecules with a particular parasite in two wild primate populations (Schwensow et al. 2010a,b). The role of MHC in the immune response to *Ascaris* has also been described in mice and rats, supporting the strong selection pressure exerted by these parasites (Kennedy et al. 1990). *Ascaris* sp. antigens, even if they stem from different species, are very similar causing similar immune reactions (Kennedy 2000). Similar to previous studies in other mouse lemur populations, allele-specific effects seemed to be more important than a “heterozygote advantage” (Schad et al. 2005; Schwensow et al. 2010a,b). Interestingly, the hybrids had a significantly higher rate of nonsynonymous substitutions in the functionally important antigen-binding sites compared with the pure species which can be a hint toward higher pathogen-driven selection pressure. However, the hybrids also revealed a significant heterozygote deficit and allelic richness was significant lower than in the pure species which might be explained by selection against hybrids with certain genotypes. It has been shown that in tension zones, heterozygote deficiency results from a balance between endogenous selection against hybrids and immigration of both parent species (Barton & Hewitt, 1985).

In the investigated mouse lemur hybrid zone, there is an evidence for bidirectional hybridization although the hybrids are morphologically more similar to *Mm* than to *Mg* (Rakotondranary et al. 2011a) and most hybrids carry a *murinus*-type mitochondrial haplotype (Gligor et al. 2009; Hapke et al. 2011). This has been interpreted as a consequence of the ongoing rapid climate change in southern Madagascar (Hannah et al., 2008) where mesic forests become drier and dry adapted species (*Mg*) expand their range into forests previously used by species (*Mm*) adapted to the more mesic environment (Lahann et al. 2006; Gligor et al. 2009; Hapke et al. 2011; Blair et al. 2014). As males are the dispersing sex in mouse lemurs (Mittermeier et al. 2010), dispersing male *Mg* moving into previously mesic forests are more likely to sire hybrids than male *Mm*.

Female *Mm* might also chose less parasitized *Mg* males (Schwensow et al. 2008a). Parasite load in *Mg* is extremely low, also in comparison to other mouse lemur studies (Schad et al. 2005; Schwensow et al. 2010a,b). Disassortative mate choice leads to an optimization of the genetic constitution of offspring in terms of parasite resistance. MHC-dependent mate selection to improve the genetic diversity of offspring has explicitly been shown in lemurs, including the study species *Microcebus murinus* (Schwensow et al. 2008a,b). While in *Mm* both sexes do not show any morphological differences irrespective of habitat, or living in sympatry or allopatry, *Mg* males have a lower body mass and shorter head–body length when living in sympatry with *Mm* than when living in allopatry. Accordingly in the hybrid zone, the average body mass of hybrids (females: 53.7 ± 3.6, males: 56.7 ± 5.7) is between those of *Mg* (females: 51.0 ± 3.6, males: 47.0 ± 4.7) and *Mm* (females: 63.8 ± 12.6, males: 56.5 ± 5.5) (Rakotondranary et al. 2011a). The high proportion of *Mm* mitochondrial haplotypes in hybrids implies that some *Mm* females prefer smaller (but less parasitized) *Mg* males over larger *Mm* males. This is an interesting trade-off as many studies have shown strong sexual selection and associated mating preferences for both, larger than average body size (e.g. Poulin and Thomas 1999) and low parasite burden (Hamilton and Zuk 1982).

## Conclusions

Despite evidence for the functional importance of MHC diversity in parasite resistance in all *Microcebus* spp., the higher parasite load in hybrids indicates that introgressive hybridization does not lead to beneficial adaptations between species and emphasize the role of parasites in maintaining diversification. Thus, in *Microcebus*, hybridization seems not to enhance the adaptability to parasites. Our study emphasizes the potential role of parasites in driving and maintaining microendemic species borders. It contributes to our understanding of one of the core questions in evolutionary biology and conservation genetics, what drives the adaptability of wildlife populations to changing environmental conditions on the one hand but limits hybridization on the other hand.
